# Supplementary material for: Comprehensive Analyses of SARS-CoV-2 Transmission in a Public Health Virology Laboratory
Source: Viruses. 2020 Aug 5;12(8):854. doi: 10.3390/v12080854 (PMC7472171; doi:10.3390/v12080854)
Supplement: Supplementary file 1 [file viruses-12-00854-s001.pdf]

viruses

MDPI

Supplementary Materials: Comprehensive Analyses of SARS-CoV-2 Transmission in a Public Health Virology Laboratory

Table S1. mixed-nucleotide mutations.

Mut Pos.	Gene	Mix %
	**S1**	
22977	spike	T (71%, 5), C (29%, 2)
22932	spike	G (85%, 11), C (15%, 2)
	**S3**	
11842	nsp6	G (80%, 21951), T (20%, 5618)
17435	nsp13	G (76%, 16558), C (24%, 5121)
19406	nsp14	G (81%, 180), C (19%, 41)
22979	spike	T (71%, 5), C (29%, 2)
25202	spike	T (76%, 23060, C (24%, 7152)
28290	nuclecapsid	T (54%, 35324), C (45%, 29474)
29091	nuclecapsid	C (72%, 21499), T (28%, 8206)

Mixed-nucleotide differences identified in samples S1 and S3. The nucleotide position, gene and mix % are indicated for each mutation.

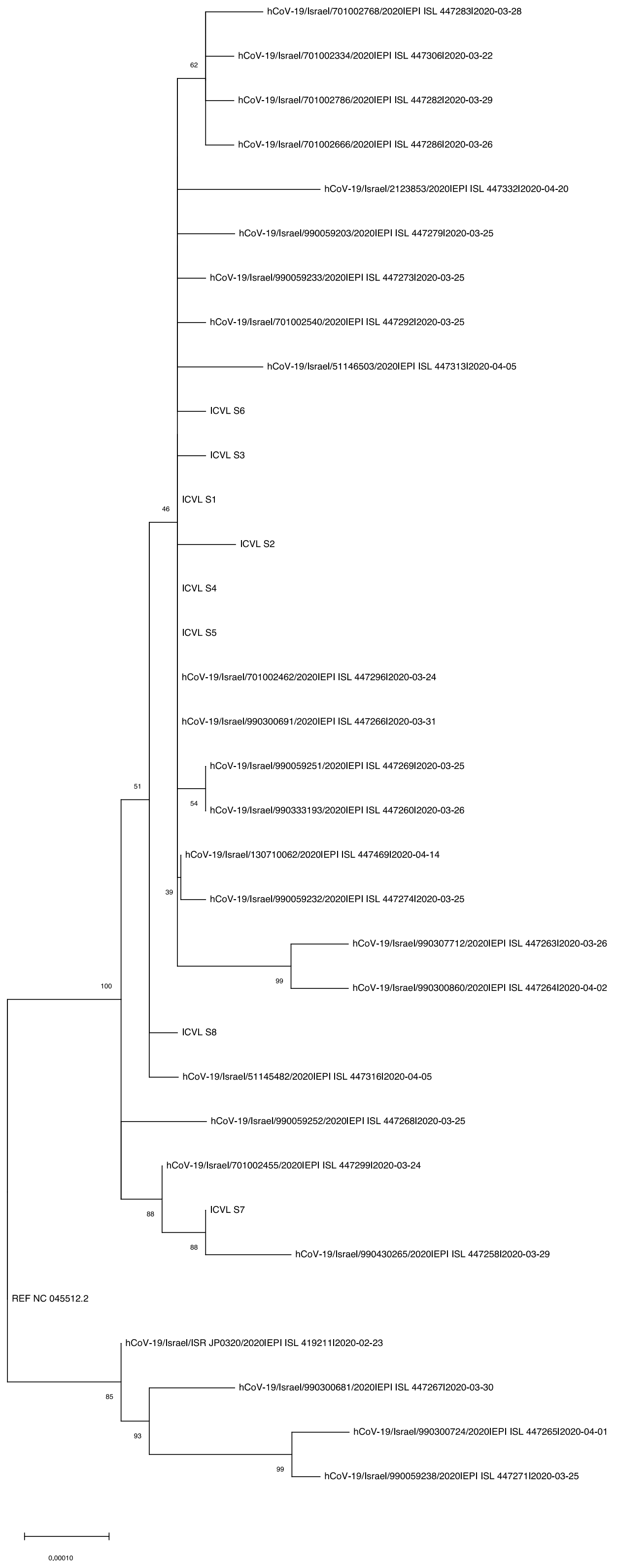

Figure S1. Whole genome-based phylogenetic tree of ICVL local outbreak and additional Israel-based samples. Phylogenetic tree of six ICVL samples (S1–6), two family-related samples (S7–8), SARS-CoV-2 reference sequence (NC 045512.2) and 25 randomly chosen Israel-based samples downloaded from GISAID. Tree was inferred by maximum likelihood based on the GTR + I + G evolutionary model. The robustness of branching pattern was tested by 1000 bootstrap replications

and the percentage of successful bootstrap replicates is indicated at the nodes, where only values of >60% are indicated.
